# Language Processing in Posterior Fossa Tumour Patients: Psycholinguistic Insights into the Word-Finding Ability

**DOI:** 10.1007/s12311-026-02026-x

**Published:** 2026-05-21

**Authors:** Rida Ahmed, Aliene Reinders, Cheyenne Svaldi, Annet Kingma, Karin Persson, Ditte Boeg Thomsen, Jonathan Kjær Grønbaek, Aske Foldbjerg Laustsen, René Mathiasen, Barry Pizer, Kristian Aquilina, Greg Fellows, Ian Kamaly, Donald Macarthur, Roel Jonkers, Marianne Juhler, Vânia de Aguiar

**Affiliations:** 1 European Master’s in Clinical Linguistics (EMCL++), University of Groningen, Groningen, The Netherlands; 2 Center for Language and Cognition Groningen (CLCG), University of Groningen, Groningen, The Netherlands; 3 Behavioral and Cognitive Neuroscience research school (BCN), University of Groningen, Groningen, The Netherlands; 4 Department of Language and Cognition - Psychology and Language Sciences, University College London, London, UK; 5 Department of Pediatrics/Pediatric Oncology, University Medical Center Groningen, Groningen, The Netherlands; 6 Department of Health Sciences, Lund University, Lund, Sweden; 7 Department of Nordic Studies and Linguistics, University of Copenhagen, Copenhagen, Denmark; 8 Department of Paediatric and Adolescent Medicine, Rigshospitalet University Hospital, Copenhagen, Denmark; 9 Department of Neurosurgery, Copenhagen University Hospital Rigshospitalet, Copenhagen, Denmark; 10 University of Liverpool, Liverpool, UK; 11 Department of Neurosurgery, Great Ormond Street Hospital, London, UK; 12 Department of Paediatric Neurosurgery, Bristol Royal Hospital for Children, Bristol, UK; 13 Royal Manchester Children’s Hospital, University of Manchester, Manchester, UK; 14 Department of Paediatric Neurosurgery, Queen’s Medical Centre, Nottingham, UK; 15 Department of Radiation Oncology, University Medical Center Groningen, Groningen, The Netherlands

**Keywords:** Infratentorial neoplasms, Posterior fossa tumors, Language disorders, Psycholinguistics, Semantics, Posterior fossa syndrome

## Abstract

**Background:**

Word finding - the ability to retrieve and produce appropriate words in response to prompts or visual stimuli - is impaired in some patients with a posterior fossa tumour. Yet, few studies use preoperative assessment as a baseline, and an in-depth linguistic analysis of tasks assessing word-finding ability remains limited. The current study aims to fill this knowledge gap by analysing pre- and postoperative word-finding ability and identifying its linguistic predictors.

**Method:**

38 English-speaking patients (19 males and 19 females), aged between 2,5 and 17,6 years and diagnosed with posterior fossa tumours were assessed before and after surgery. Performance was assessed using a picture-naming task, Wordrace, measuring both accuracy and reaction times. These measures were interpreted in terms of their correlation with linguistic levels (i.e., lexical, semantic, phonological).

**Results:**

Patients exhibited a significant slowing in word-finding speed following surgery, while accuracy remained stable across assessment points. Despite this decline in speed, we did not find evidence for a change in the influence of psycholinguistic factors on word-finding ability in our sample. Lexical-semantic variables predicted word-finding speed, whereas accuracy was influenced only by lexical variables.

**Conclusion:**

The findings suggest that although general performance declined postoperatively, we did not find evidence in our sample of disruption to the underlying linguistic processes engaged during word-finding. However, the absence of control group limits interpretation, and future studies comparing patients with healthy controls may reveal more subtle differences. The study emphasises the importance of longitudinal assessment in patients with posterior fossa tumours and future studies should incorporate additional timepoints to examine the potential effects of radiotherapy and chemotherapy on word-finding over time.

## Introduction

 Central nervous system tumours are the second most common group of childhood neoplasms after leukaemia and continue to cause the highest number of childhood deaths related to cancer [1]. These statistics still hold even though survival rates of children with cancer have increased significantly over the last few decades [2]. Around half of childhood brain tumours arise in the posterior fossa that contains the cerebellum and the brainstem [3]. With improving treatment and care standards, it is important to ensure that survivors lead socially engaged lives with minimal functional impairments. Children with posterior fossa tumours (PFTs) present with language impairments at all processing levels, from producing individual words to forming sentences and using language appropriately in social situations, prompting further investigation into the nature of these impairments [4]. Since speech and language impairments create a barrier to effective social engagement [5], investigating important skills such as word-finding abilities is useful in gaining insights into language deficits affecting different linguistic processes [6, 7]. The current study explores the nature of language processing underlying word-finding abilities in the PFT population using a picture-naming task. Furthermore, it examines potential changes in the factors influencing naming performance, measured before and after tumour surgery.

### The Linguistic Cerebellum

The cerebellum has traditionally been attributed a role in motor function [8]. Nonetheless, by now, many studies using clinical and neuroimaging methods, mostly with adults, have confirmed its role in cognitive functions, including a variety of language functions, indexed by tasks such as verbal fluency [9, 10], lexical decision [11], verb generation [12], and reading aloud [11]. Specific to lexical access - a process of retrieving words from the mental dictionary, right cerebellar involvement has been shown in phonemic fluency [13] and lexical decision [11]. The right cerebellum also showed activation when participants were asked to pay attention to the semantic relations between words [14]. Moreover, a recent study showed that electrical stimulation of the posterior cerebellum bilaterally facilitated the retrieval of semantically related words, confirming the cerebellum’s role in word retrieval in healthy participants [15]. In addition, both hemispheres of the cerebellum have shown to be sensitive to distinct word properties during spoken language comprehension [16]. Specifically, higher word frequency was positively associated with activation in bilateral Crus I (extending into Crus II - subregions in the lateral posterior cerebellum), and superior lobule VI and IX. Moreover, lower phonological neighbourhood density (i.e., fewer similar-sounding words) were linked to greater activation in bilateral Crus I/II regions, with a stronger effect in the right hemisphere.

### Impairments in Children with PFTs

Surgical resection of a PFT may be followed by an array of motor, linguistic, and neurobehavioral/affective symptoms [17]. These have been collectively named ‘post-operative pediatric cerebellar mutism syndrome (ppCMS)’ [18], or Posterior Fossa Syndrome (PFS) [19]. A hallmark of this syndrome is a temporary period of absence of speech (mutism, PFS1) or severely reduced speech (PFS2), which may occur on its own or follow a period of mutism; together, these are referred to as post-operative speech impairment (POSI) [20, 21]. Young age at diagnosis, high-grade tumour, and central midline location lead to an increased risk of developing POSI.

Importantly, not all post-surgical impairments fall under the formal diagnosis of PFS/pCMS. Even in the absence of mutism, children may show fine and gross motor impairments [22] and cognitive sequelae, for example, low IQ, and poor memory, attention, and executive functioning [23]. Dysarthria, especially of the ataxic type, is also commonly reported, whether or not mutism is present [24, 25].

Furthermore, several studies have reported impairments in language abilities after surgery, regardless of whether the child experienced a phase of mutism or reduced speech [4, 26]. Yet, this literature is characterized by a great inconsistency, as some studies did not report group differences in language ability when comparing groups of PFT survivors and healthy controls (see [27, 28]). A recent systematic review suggests that this inconsistency may partly reflect the substantial inter-individual variability in which aspects of language are impaired, as well as substantial methodological differences across studies, highlighting the need to study language in greater granularity with precise methods and attention to underlying linguistic mechanisms [4]. This systematic review of individuals reported in the literature showed that PFT survivors may show varying language impairment profiles, with individuals presenting many possible combinations of impaired and spared ability across levels of language processing [29, 30], for example in semantics (knowledge of meanings) compared to morphosyntax (grammar), or pragmatics (language use in context).

#### Word-Finding Difficulties in the PFT Population

When looking more specifically at studies examining word-finding abilities (see Table 9 in the Appendix for a summary of the literature), eight studies found significantly low performance of PFT patients in expressive vocabulary and word-finding tests [26, 35, 37, 40, 44, 45, 48], four showed patients’ performance comparable to control participants or norms [28, 36, 42, 47], and the remaining ten had their results complicated due to various factors (for example, no control comparison [31]; merging naming scores with other tests to report a single measure of verbal intelligence [34]. Thus, the literature is divided on the lexical retrieval abilities in PFT patients, mainly because of a lack of experimental control. This can be expected in patient populations like these since testing specific language functions is neither practical due to fatigue and affective symptoms nor a priority. While these studies focused on whether word-finding deficits were present, one recent study investigated the nature of errors produced by children, providing insight into what may go wrong in language processing, leading to poor performance [48]. Their error analysis found that PFT survivors mostly produced semantically side-oriented errors, that is, a closely related word in meaning (e.g., pear instead of apple).

#### The Pre- vs. Post-surgical Onset of Impairments

Although the studies described above report on post-surgical impairments, children with PFTs may show impairments due to the presence of the tumour itself (before surgery), and also as a consequence of the necessary surgery and post-surgical treatments (i.e., radio- and/or chemotherapy). For instance, one study found that all patients who developed CMS after surgery had pathological naming preoperatively [37]. Another study reported slow (37%), inaccurate (24%), or slow and inaccurate (16%) word finding in PFT survivors already before surgery [48]. Moreover, when analysing word-finding abilities of PFT patients both before and after surgery, patients were found to be substantially impaired in comparison to controls in terms of naming speed at both timepoints [49]. Upon close inspection of the individual data, variability is reported such that some patients’ word-finding ability worsened after surgery while it improved for others.

Importantly, the causal nature of word-finding abilities might change from pre- to post-surgery. First, impairments may exist because the tumour affects the anatomical substrates that have a role in word finding but depending on tumour type (low-grade or high-grade), this may happen at varying speeds, sometimes allowing for the language system to adapt to the anatomical changes via neuroplastic mechanisms [50]. This disruption may occur locally within the cerebellum due to its contribution to language processing [51], or through diaschisis, where tumour-induced compression causes disrupted connectivity with supratentorial language regions [52, 53]. In addition, language impairment can result from local disruption to supratentorial areas themselves, due to tumour-related hydrocephalus [54]. On the other hand, surgery, while necessary for survival, causes a sudden, acute lesion as well as post-surgical edema in the posterior fossa region, which affects structures beyond the tumoral tissue, potentially affecting additional language processes. Hence, the neurocognitive nature of the word-finding difficulty may change from pre- to post-surgery.

### The Chain of Processes in Word Finding

**Fig. 1:**
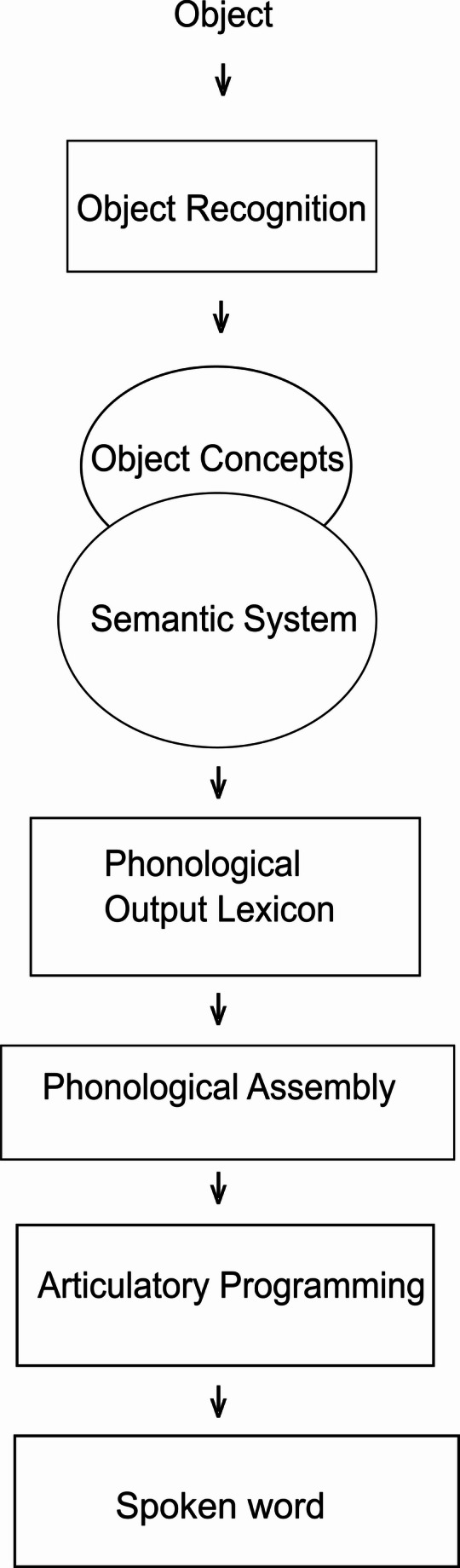
Language processing model for production of single words. Adapted from [56]

Psycholinguistic models of language processing suggest that word retrieval may fail or become challenging due to language processing impairments, which can happen at different stages of the word retrieval process. As illustrated in Fig. 1, model adapted from [55, 56], picture naming comprises different sequential processes: *Object Recognition* is initiated when a person sees an object in a picture or in real life. Then follows non-verbal conceptual information about the object, such as knowing that an apple is something to eat, accessed through the level called *Object Concepts*. The information is then processed in the *Semantic System*, which is defined as the elements of knowledge that collectively compose the meaning of words.

The next stage in the word retrieval process is the retrieval of the spoken word forms from the *Phonological Output Lexicon*, where known vocabulary is stored as combinations of speech sounds. Once a phonological form has been activated from the output lexicon, a phoneme string is generated by the *Phonological Assembly*. Finally, the phonemes from this sequence are fed into the *Articulatory Programming* stage, which generates neuromuscular commands from these phonemes, and the word is ultimately articulated.

Factors that influence speed and accuracy of naming in individuals with language disorders have received much attention in previous literature [57, 58]. Among these factors are the psycholinguistic properties of words that are to be named, such as word frequency, age of acquisition, and familiarity. Some of them are said to accelerate the retrieval of words (i.e., word frequency [59]; age of acquisition [60]), while others slow it down (i.e., phonological neighbourhood density [61]; word length [62]). This effect of word properties on lexical retrieval can be explained by the differential involvement of each language processing level (e.g., semantics, phonology) in language production. Importantly, the critical variable framework postulates that when a given variable affects performance in word retrieval, it is considered evidence of impaired language processing at the level of functioning relating to that specific variable [63].

In what follows, variables associated with specific levels of language processing shown in Fig. 1 will be described. It should be noted that the assignment of psycholinguistic variables to specific language processing levels is not always consistent. For example, the effects of age of acquisition of a word could be of a lexical origin (increased exposure to a word helps in its fast retrieval) or a semantic origin (early acquired words are processed quicker because they are positioned more centrally in the semantic network) [64]. The following section outlines the most commonly reported associations in the literature.

#### Semantic Variables

Several semantic variables have been found to influence the lexical retrieval process at the Semantic System level shown in Fig. 1. For instance, imageability, which refers to the extent to which a word evokes a mental image, has been shown to facilitate word retrieval in both neurotypical individuals and people with aphasia [65–67]. Similarly, concreteness, which is defined as the degree to which a noun can be touched or visualized, supports the word retrieval process by creating strong semantic associations [68]. The number of semantic features, defined as the number of distinct conceptual attributes associated with a word, also facilitates naming speed and accuracy in neurotypical individuals by increasing semantic and lexical activation [69, 70]. It has also been found to be a key predictor of naming performance in people with aphasia [71].

#### Lexical Variables

The lexical level of processing is reflected in the Phonological Output Lexicon in Fig. 1 and is influenced by variables relating to the way words are organized in the mental lexicon. One such variable is word frequency. It refers to how often language users are likely to encounter a word in everyday use and has a well-established facilitatory effect on naming in neurotypical individuals [72–75]. Secondly, age of acquisition (AoA) of a word also affects naming in neurotypical individuals, with early-acquired words retrieved faster [76–79]. Word familiarity is a more subjective measure of how often a person encounters a word - calculated based on participants’ ratings of how familiar a word feels to them - and it correlates strongly with word frequency and similarly facilitates naming [78, 80, 81]. It has also been shown to distinguish between variants of primary progressive aphasia [67]. Lastly, the phonological neighbourhood density (PND) is the number of words that can be formed by replacing, deleting, or adding one phoneme to the original word [82]. It is important to note that, although based on sound similarity, phonological neighbourhood density is considered a lexical-level variable because it reflects how words are organized in the mental lexicon [83]. It facilitates naming in people with aphasia [84], but shows variable effects in children depending on language development status [85]. These effects are often explained by interactive activation models, where activation spreads between phonemes and words, resulting in either competition or facilitation [82, 86].

#### Phonological Variables

Phonological variables like word length influence lexical retrieval at stages prior to articulation. Specifically, the phonological output buffer precedes articulatory programming, temporarily storing speech sounds. Word length is measured by either the number of phonemes or syllables in a word. In some populations, such as people with aphasia, longer words result in more errors in naming [66], although a reverse effect has also been reported where longer words are more easily retrieved due to fewer phonological neighbours [87]. Specifically, longer word length in syllables has been shown to correlate with longer naming latencies in individuals with developmental language disorder [88]. Lastly, the number of consonant clusters in a word affects the Articulatory Programming stage of word production [89]. The increase in the number of consonant clusters has been associated with a higher number of errors in naming in children with DLD [90, 91].

Previous studies with participants with language disorders have employed analysis of the psycholinguistic properties of the words produced. For instance, when checking for the psycholinguistic properties of the words produced by cerebellar tumour patients, impairments were observed at multiple levels of language processing [92]. Similarly, in spontaneous speech in bilingual children with developmental language disorder, it was found that the difference in phonological neighbourhood density between nouns and verbs was more pronounced in sequential bilingual children with DLD than in typically developing children [93]. Using a similar approach, a study investigating naming in individuals with aphasia found that the ‘lexical usage’ factor (composed of word frequency, familiarity, and age of acquisition) significantly differentiated between patients with fluent and non-fluent aphasia regarding naming accuracy [94]. Lastly, a role of word familiarity has been reported in driving naming performance in patients with primary progressive aphasia [67].

### Present Investigation

#### Research Gap

Not only is the previous literature divided on the presence/absence of word-retrieval deficits in PFT patients, but the linguistic nature of impairments behind these deficits has been scarcely investigated. Psycholinguistic properties of words provide a window to investigate the processing dynamics that may be related to the performance in word finding even when the word is ultimately named correctly (e.g., through relations with reaction times (RTs)). This has not been explored in previous studies with the PFT population. Clinically, such an investigation is significant for two reasons: (1) identifying whether deficits stem from semantic, lexical, or phonological stages can inform targeted therapies (e.g., semantic feature training vs. phonological cueing), (2) subtle psycholinguistic effects (e.g., slowed RTs for low-frequency words) may serve as markers of cerebellar-cortical circuit disruption in patients with otherwise intact accuracy, potentially identifying cases at risk of presenting worse language performance.

Moreover, the early postoperative stage in patients who undergo surgery in supratentorial language-eloquent areas is characterised by a decline in all language functions, including those involved in lexical retrieval [95, 96]. This is attributed to the significant tissue damage as a result of surgery. Since several studies have identified a role of the cerebellum in language, and lesions in this region have been associated with subsequent language impairments [97], the current study also explored whether linguistic processing behind word finding in PFT patients is affected by the tumour surgery by comparing performance between the preoperative and the early postoperative periods.

#### Research Questions and Hypotheses

The current study aims to address three main questions.

First, we examine whether word-finding ability changes from before to after surgery in patients with PFTs. It is expected that both the speed and accuracy of word retrieval will decline postoperatively, reflecting the potential impact of tumour removal on neural structures involved in language processing.

Second, we explore which underlying linguistic processes drive word-finding abilities in the PFT patients. Based on prior findings, such as the prevalence of semantically related errors in PFT patients [48] and reports of semantic paraphasias in cerebellar stroke patients [97] - we hypothesize that semantic and lexical-semantic levels will play a primary role in driving word-finding performance. The exploration for the rest of the linguistic levels is exploratory.

Finally, the study investigates whether the underlying linguistic processes differ in predicting word-finding abilities pre- and postoperatively in PFT patients. We anticipate that damage to brain tissue following resection may alter the relative influence of psycholinguistic levels on word-finding ability, indicating a shift in the mechanisms supporting word-finding ability.

## Method

### Participants

Thirty-eight English-speaking patients, 19 males and 19 females from the United Kingdom, included in the European Study of the Cerebellar Mutism Syndrome [98] formed the participant group for the current study. The European Study of CMS is a large multicenter study initiated in 2014 to investigate the incidence, symptoms, risk factors, and prognosis of CMS in children following surgery for posterior fossa tumours. The patients included in the current sub-study did not present with a history of developmental language disorder (DLD) or other speech-language disorders. Table 1 presents the demographic, tumour, and postoperative speech characteristics of participants.

**Table 1:** Demographic, tumour, and postoperative speech characteristics of participants (*n* = 38)

Characteristic	Included cohort (*n* = 38)
Sex	19 males (50) 19 females (50)
Age at surgery (years)	Mean = 8,8 Range = 2,5–17,6 SD = 4
Tumour histology	Pilocytic astrocytoma: 20 (≈ 53) Medulloblastoma: 8 (≈ 21) Ependymoma: 6 (≈ 16) Ganglioglioma: 1 (≈ 3) Unknown: 3 (≈ 8)
POSI status	Habitual speech: 30 (≈ 79) Mute: 4 (≈ 10) Reduced speech: 2 (≈ 5) Unknown: 2 (≈ 5)

*POSI,* Postoperative speech impairment. Categorical variables in n (%)

### Materials

The study used audio recordings of the Wordrace task to determine accuracy and reaction times. Wordrace is a picture-naming task especially designed for the European study of CMS to test word-finding ability [99]. It contains 25 pictures that are shown either on screen or on paper one by one, and which need to be named as fast as possible. The test has been normed for the Swedish language and has shown a high test-retest reliability for measuring accuracy (*r*=0.894) and speed (*r*=0.627) [100]. It is a good alternative to traditional naming tests as it puts minimal demands on executive functioning, thus helping in measuring just the word-finding speed and accuracy [48]. For some target words, alternatives are accepted: for instance, ship for boat and chicken for rooster.

### Procedure

The study used retrospective Wordrace data collected from patients at UK sites participating in the European Study of CMS, including Alder Hey Children’s Hospital (Liverpool), Great Ormond Street Hospital (London), and centers in Manchester, Bristol, and Nottingham. We used data from two assessment points, i.e., preoperatively (assessment point 1) and 1–4 weeks postoperatively (assessment point 2). The tester explained the task to the participants before administering it and then turned the pages or swiped on the tablet screen after the participant named each picture. As per the instructions, if a picture was not named after 5 s, the tester was supposed to move on to the next one, but this was not followed in all the testing sessions. The tester also kept track of the total time taken to complete the test with a stopwatch. Only minimal feedback was provided, such as ‘good job’, or the tester asked ‘what’s that?’

### Data Processing

#### Calculating Reaction Times

**Fig. 2:**
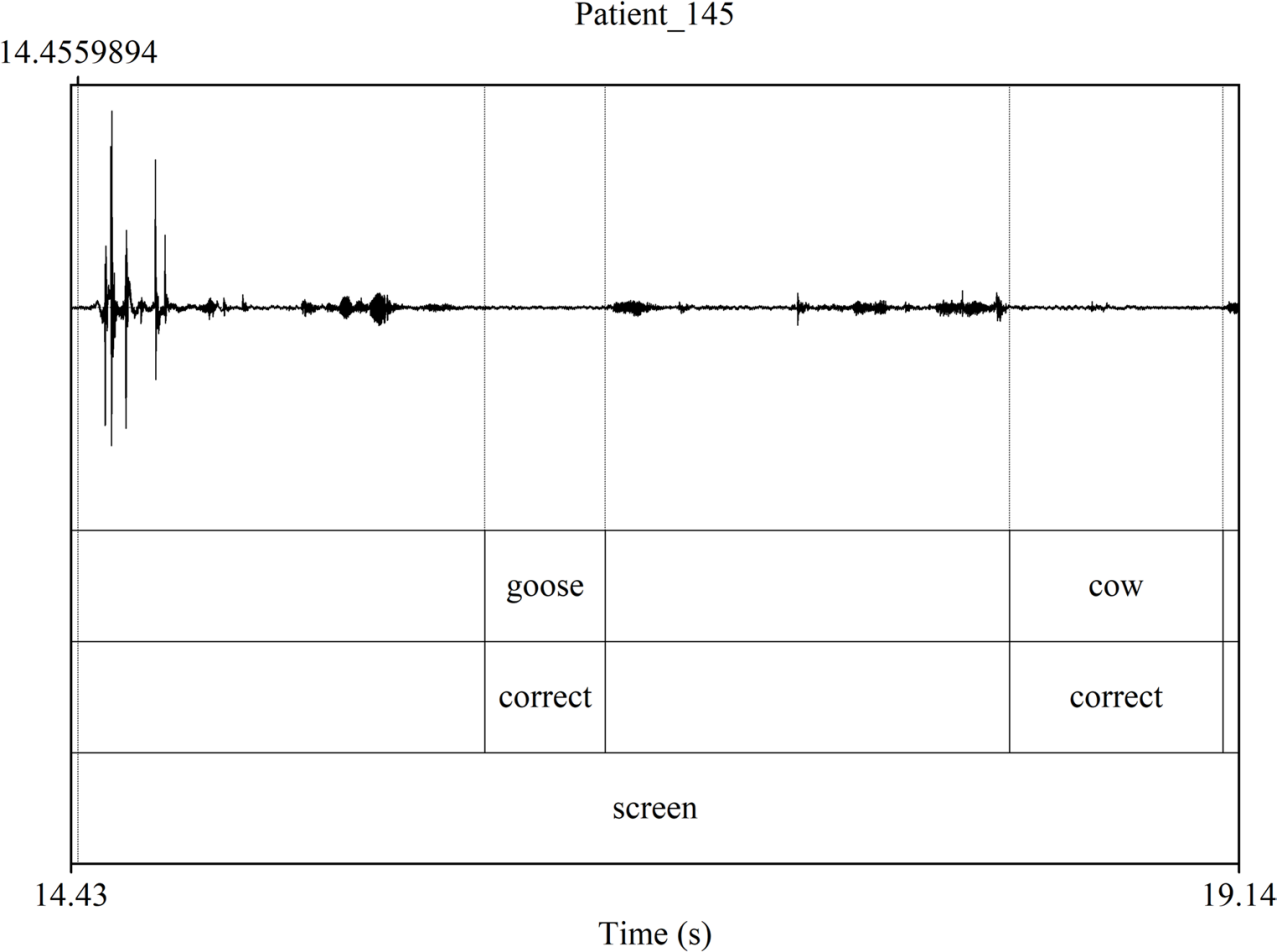
Praat TextGrid for each speech file. Note. Intervals in Tier one are labeled as target words and represent the reaction time for naming that word. The starting point of each interval in tier one is the approximate moment at which the stimulus was visible (i.e., the page-flipping sound for paper format and the key press sound for screen format). Therefore, the interval labeled *goose* in the figure is the reaction time for this item, and the uttered word comes after it

Reaction time and accuracy were determined for each item on Wordrace using Praat [101], a software used for phonetic analysis of audio data. See Fig. 2 for an example of what the TextGrid looked like for each speech sample (an interval is created just before a word is articulated and labeled for easier extraction of reaction times and accuracy for each label using Praat script). A response was considered correct if the target word was eventually named, even if the participant named a semantically related or unrelated word before naming the target word. Only correct responses were considered for reaction time analyses. Since the database only had audio recordings and the task was administered in different formats (screen or paper) in different centres across the UK, we defined specific markers of the start of the reaction time: an audible tap on the keyboard by the examiner for centres that conducted it on screen and the start of page flipping sound for centres that conducted it on paper. A tap or page flip was not audible for some audio recordings that probably administered the task on a tablet. For these cases, the pause between the naming of the previous item and the next item served as the reaction time. These different modes of task administration were adjusted for later in the analysis. The endpoint of the reaction time was marked at the onset of the acoustic marker of the first phoneme produced in the response (voicing for voiced consonants and vowels; or, for example, the onset of the plosive or fricative signal), as soon as this was visible in the waveform corresponding to the participant starting to name the picture. This ensures we only measured the time the participant took to retrieve the word, not other unrelated delays, such as phoneme elongation or interrupted articulation.

Some participants named the pictures starting with the articles ‘a’, ‘the’ and ‘an’ while some started with the word ‘some’, such as ‘some bread’. Reaction time was noted after the article or the quantifier since participants may elongate these when struggling to retrieve words. Moreover, if the participant hesitated to say a word by articulating the first phoneme and then pausing and saying the whole word, the reaction time was noted until they said the word itself. In some cases, the participant said a semantically related word before ultimately naming the target word. In such cases, reaction time was noted until the target word was named. Sometimes, the participants coughed while naming a picture. Their reaction time was noted before they began coughing only if they had already articulated 20% of the phonemes of the word. Otherwise, the item was not considered for reaction time. The items for which the child could only say the target word upon hearing it from the tester were excluded and marked as ‘no response’.

#### Coding Words for Psycholinguistic Properties

To answer research questions 2 and 3, the study considered psycholinguistic word property norms for British English. Table 2 shows databases that are specifically normed for British English that were used for the present study. Consonant clusters and word length in syllables were defined manually. Each word in the Wordrace test was coded for each word property. Table 3 shows some examples of psycholinguistic word properties of items in Wordrace.

**Table 2:** Psycholinguistic properties of words

Word property	Database	Linguistic level	Rating scale
Word frequency	SUBTLEX-UK [102]	Lexical	Zipf scale (1–7) 1–3: Low frequency 4–7: High frequency
Phonological neighbourhood density	CLEARPOND (EnglishPOND) [103]	Lexical	Number of phonological neighbours after adding/deleting/substituting 1 phoneme
Age of acquisition (AoA)	The Bristol norms for age of acquisition [104]	Lexical	Average AoA in years across participants
Semantic features	Semantic feature production norms [69]	Semantic	No. of distinct features listed for each concept
Concreteness	The Glasgow Norms [105]	Semantic	1–7 scale (1 = concrete, 7 = abstract - we used mean concreteness rating across participants)
Imageability	The Glasgow Norms [105]	Semantic	1–7 scale (1 = very unimageable, 7 = very imageable)
Familiarity	The Bristol norms for familiarity [104]	Semantic/lexical	1–7 scale (1 = very unfamiliar, 7 = very familiar)
Length in phonemes	CLEARPOND (EnglishPOND) [103]	Phonological	No. of phonemes
Length in Syllables	Self-determined	Phonological	No. of syllables
Consonant clusters	Self-determined	Phonological	No. of consonant clusters

**Table 3:** Examples of psycholinguistic properties of items in the Wordrace

Noun	Frequency	Familiarity	Concreteness	Imageability	Phonological neighbourhood density	Semantic features
Goose	4.027770003	5.625	4.81	6.2941	19	1.909
Dice	3.708994391	4.9167	4.86	6.3704	29	2.108
Cake	4.805415495	6.4839	4.81	6.6765	35	2.413
Star	5.040815253	6.3235	4.69	6.5806	17	1.377
Flower	4.501236584	6.5862	5	6.7879	5	2.15

#### Dimensionality Reduction via Principal Component Analysis

The variables in Table 2 were to be used as predictors of word-finding speed and accuracy in analyses addressing the research questions 2 and 3. However, these make a large number of predictors and our interest was in the role of language processing levels, and not specific variables. Given that several variables are expected to reflect overlapping linguistic processing levels, variables were clustered together when they could be merged. This was done using a Principal Component Analysis (PCA) with values of all included psycholinguistic variables in Table 2 for the nouns that the participants used (*n* = 40); this included all the different synonyms used. A similar approach has been used by previous studies (see for example [94]),. The PCA was done in RStudio [106]. All variables were inserted into the PCA at once (not per level of language processing). This way, their clustering was entirely data-driven and not based on previous assumptions.

**Fig. 3:**
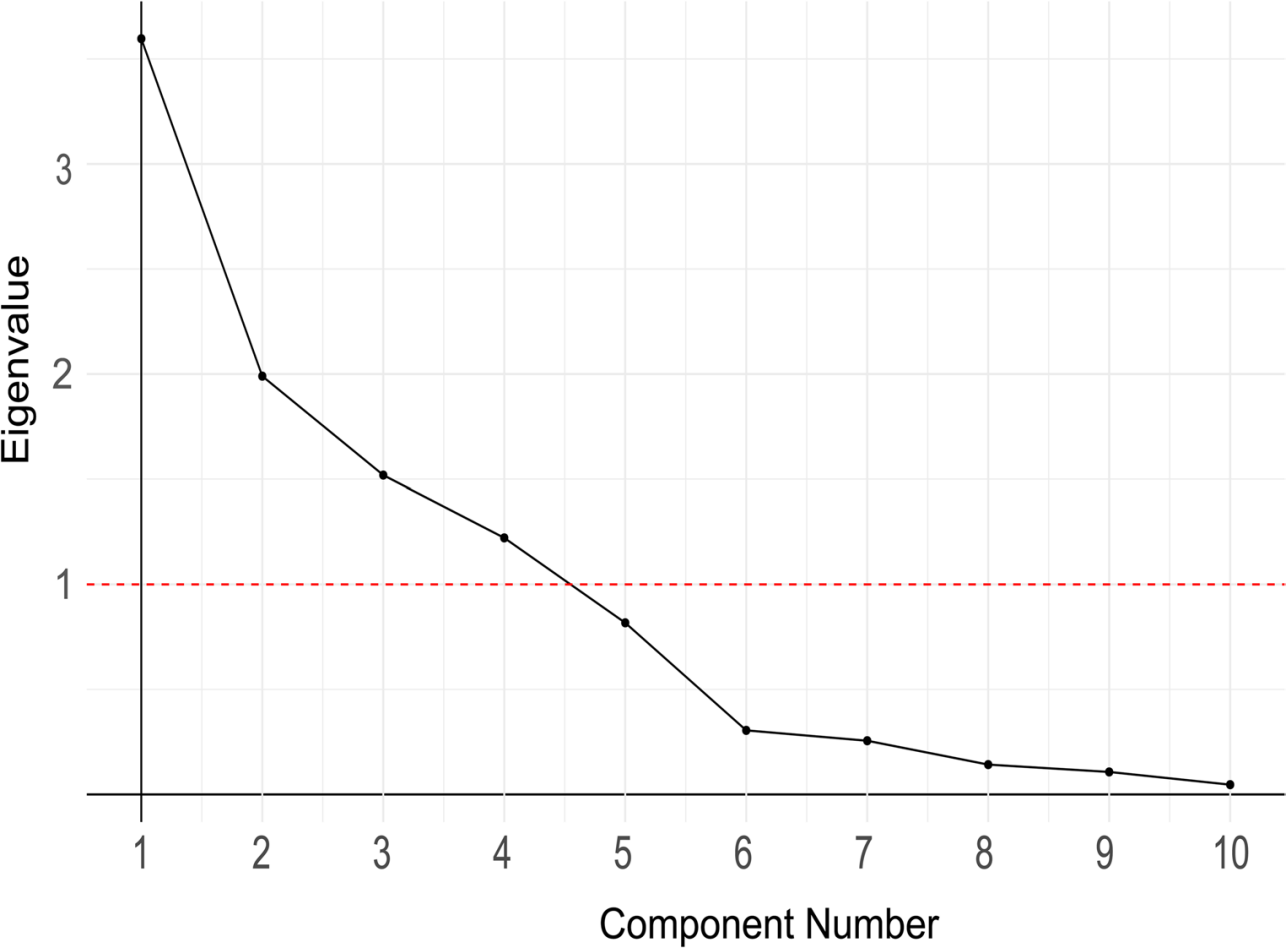
Scree plot of the principal component analysis

To prepare for PCA, all the values were first *z*-scaled to get a uniform scale because different variables were originally rated on varied scales. For example, familiarity is rated on a scale of one to seven, whereas the phonological neighbourhood density is in the form of continuous numbers, so not standardizing can result in an artificial dominance of one variable with a larger scale. The number of principal components to be retained was determined by two criteria based on previous research [92, 107], i.e., the cumulative variance explained should be more than 70%, and the components should have an eigenvalue above 1.0. The first four components explained 81.8% variance in the data; all of them have an eigenvalue higher than 1.0. See Table 4 for the loadings of different variables on the components where a value equal to or higher than 0.45 is considered a meaningful contribution and is bolded. See Table 5 for the explained variance and Fig. 3 for the scree plot for eigenvalues of components.

**Table 4:** Component loadings

	C1	C2	C3	C4
Length in syllables	−0.27	**0.50**	0.23	0.24
Length in phonemes	**0.87**	−0.3	−0.12	−0.17
Phonological neighbourhood	**−0.88**	0.07	0.05	0.23
Consonant clusters	**0.95**	0.02	−0.04	0.01
Imageability	−0.03	**0.96**	0.15	−0.06
Semantic features	−0.13	0.10	−0.10	**0.92**
Age of acquisition	0.24	−0.14	**−0.84**	0.29
Familiarity	0.09	0.10	**0.86**	0.22
Frequency	−0.39	−0.12	**0.51**	**0.67**
Concreteness	−0.06	**0.94**	−0.06	0.01

**Table 5:** Summary

	C1	C2	C3	C4
SS loadings	2.750	2.210	1.811	1.561
Proportion variance	0.275	0.221	0.181	0.156
Cumulative variance	0.275	0.496	0.677	0.833

Based on the values of component loadings (i.e., > 0.45), three variables, namely consonant clusters, length in phonemes, and phonological neighbourhood density loaded onto the first component. This is a phonological component since all these variables are reported in the literature to relate to phonological processing, either at the level of the output lexicon or the phonological output buffer. Since these variables are separated from the more classical lexical variables (e.g., frequency), their combination into a component may reflect post-lexical phonological processing related to the functioning of the output buffer or articulatory programming and execution. The second component constitutes imageability, concreteness, and length in syllables – an unexpectedly mixed grouping that we interpret as predominantly semantic due to the strong loadings of the first two variables (i.e., 0.96 and 0.94 respectively). Next is the lexical component, consisting of age of acquisition, familiarity, and frequency. Lastly, the number of semantic features and frequency made a significant contribution to the last component reflecting a lexical-semantic component. The structure of the C4 component can be explained by the fact that the number of semantic features and frequency go hand in hand (see for example [108], about the interaction between number of semantic features and word frequency). The number of semantic features represents the variety of contexts an object can be encountered in (for example, a chicken is something you find in a farm, is a bird, is a type of food, makes a sound, can be used as a metaphor, etc.). The higher the number of semantic features of an item, the higher its frequency rating would be.

A new dataset was created by averaging the scaled values of each variable that made a significant contribution to a component. For example, the values of length in phonemes, phonological neighbourhood density, and consonant clusters of each word were averaged to attain a value for C1 - the phonological component of that word. The same was done for C2-4 in relation to the variables with meaningful contributions. Since the age of acquisition and phonological neighbourhood density had negative loadings as a result of the PCA, their values were multiplied by −1 prior to averaging, to ensure all variables contributed in the same direction. The resulting dataset was used for all subsequent analyses.

### Statistical Analyses

The statistical analyses were performed in RStudio [106] and followed three steps. Since the reaction time data from the Wordrace was acquired with a non-traditional method, we needed to establish that the test yielded expected output. Naming accuracy, speed, and lexical access efficiency all improve as children’s linguistic and cognitive systems mature. Therefore, a significant and theoretically expected correlation between test performance and age supports the anticipated directionality of the task, indicating that the test captures genuine developmental differences in word-finding ability rather than random variation or unrelated skills. Therefore, a linear regression analysis was run using age and log-transformed RTs as the predictor and outcome variables, respectively. Moreover, a logistic regression analysis was employed to see how age correlated with naming accuracy.

Second, the change in RTs and accuracy from the first assessment point to the second was investigated by running a linear mixed effects model and a generalized mixed effects model with RTs and accuracy as outcome variables, respectively, and the assessment point as the predictor variable. Both models used participants as random intercepts because we expect a lot of inter-individual variability in the severity of naming difficulty.

Third, to answer research question 2, a mixed-effects regression analysis was employed, and the correlation between principal components and RTs was investigated. Participants were added as random intercepts. Log-transformed RTs for correct responses were put as the outcome variable in the model. A separate model took naming accuracy as the outcome variable. This model employed a generalized mixed-effects regression analysis to examine the relationship between principal components and naming accuracy (1 = correct, 0 = incorrect).

Lastly, to investigate the change in the way principal components predict performance across assessment points, the study employed two mixed-effects regression analyses similar to the previous models for research question 2, but now with interaction terms between each component and each assessment point (pre- vs. post-surgery). This determined whether the correlation between a certain component and naming accuracy and/or RT depended on assessment time.

## Results

### Developmental Sensitivity of Wordrace

**Fig. 4:**
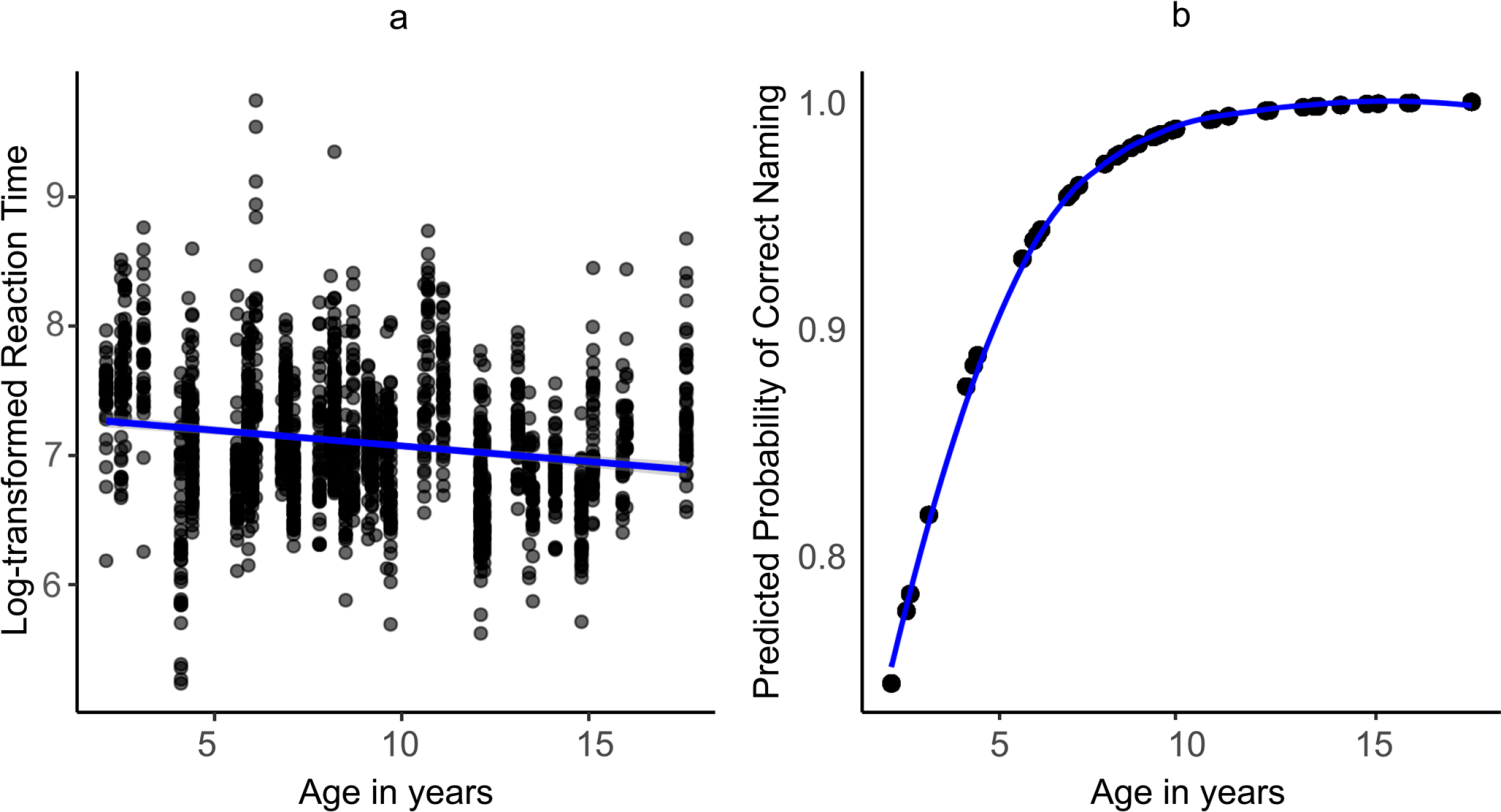
The effect of age on naming speed (**a**) and probability of correct naming (**b**)

A linear regression analysis showed a negative and significant correlation between the participants’ age and the naming reaction times (β = −0.02, 95% CI =−0.03, −0.02, *p* < 0.001), meaning that children get faster at naming pictures with increasing age. Further, the logistic regression analysis yielded a significant positive correlation between age and naming accuracy, (odds ratio of 1.55, 95% CI = 1.42, 1.70, *p* < 0.001). Figure 4a and b show these correlations.

### Change in Word-Finding Ability from Pre- to Post-surgery

**Fig. 5:**
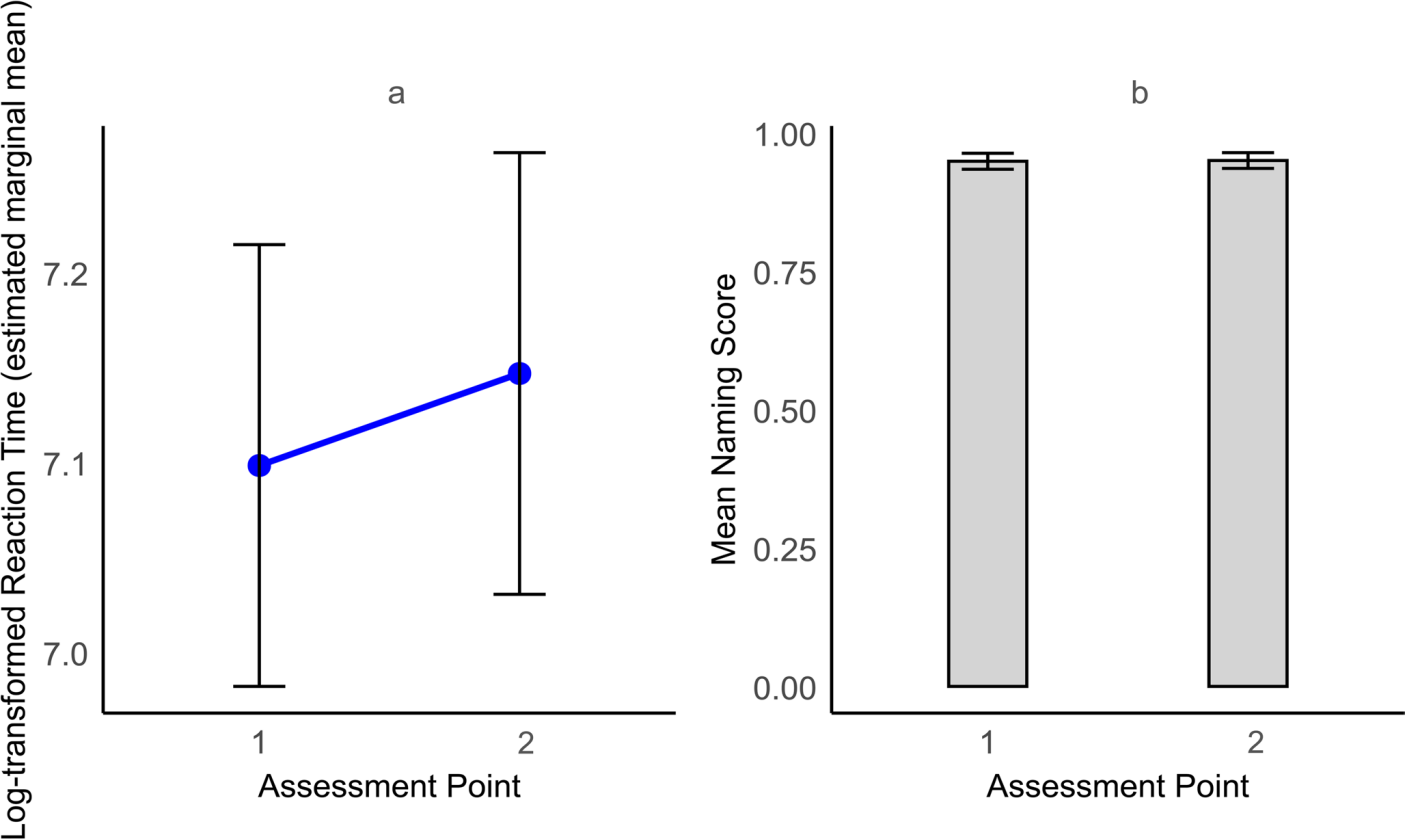
Naming speed (**a**) and accuracy (**b**) across assessment moments

Reaction times increased significantly from pre- to post-surgery (β = 0.05, 95% CI = 0.01–0.09, *p* = 0.011), while naming accuracy remained constant (Odds Ratio = 1.03, 95% CI = 0.64–1.64, *p* = 0.917), staying near ceiling levels. The estimate of 0.05 reflects the increase in log-transformed reaction times from pre- to post-surgery. When back-transformed, this corresponds to a 5.1% increase, or approximately 70 milliseconds, based on the pre-surgery mean of 1375 milliseconds. Table 6 presents the results of the mixed-effects regression analysis for reaction times and generalized mixed-effects regression for naming accuracy. Figure 5a illustrates the confidence intervals for reaction times plotted across both assessment points, and Fig. 5b features a bar graph showing that the accuracy scores remained consistently close to 1 across assessment moments.

**Table 6:** Results of mixed-effects regression for change in reaction times and generalized mixed-effects regression for change in naming accuracy across assessments

Outcome variable	Predictors	Estimates	CI	*p*
Reaction times	(Intercept)	7.10	6.99–7.21	**<0.001**
	assessment point [2]	0.05	0.01–0.09	**0.011**
		Odds ratios		
Accuracy	(Intercept)	123.64	38.09–401.31	**<0.001**
	assessment point [2]	1.03	0.64–1.64	0.917

### Language Processing Underlying Word-finding Ability

**Fig. 6:**
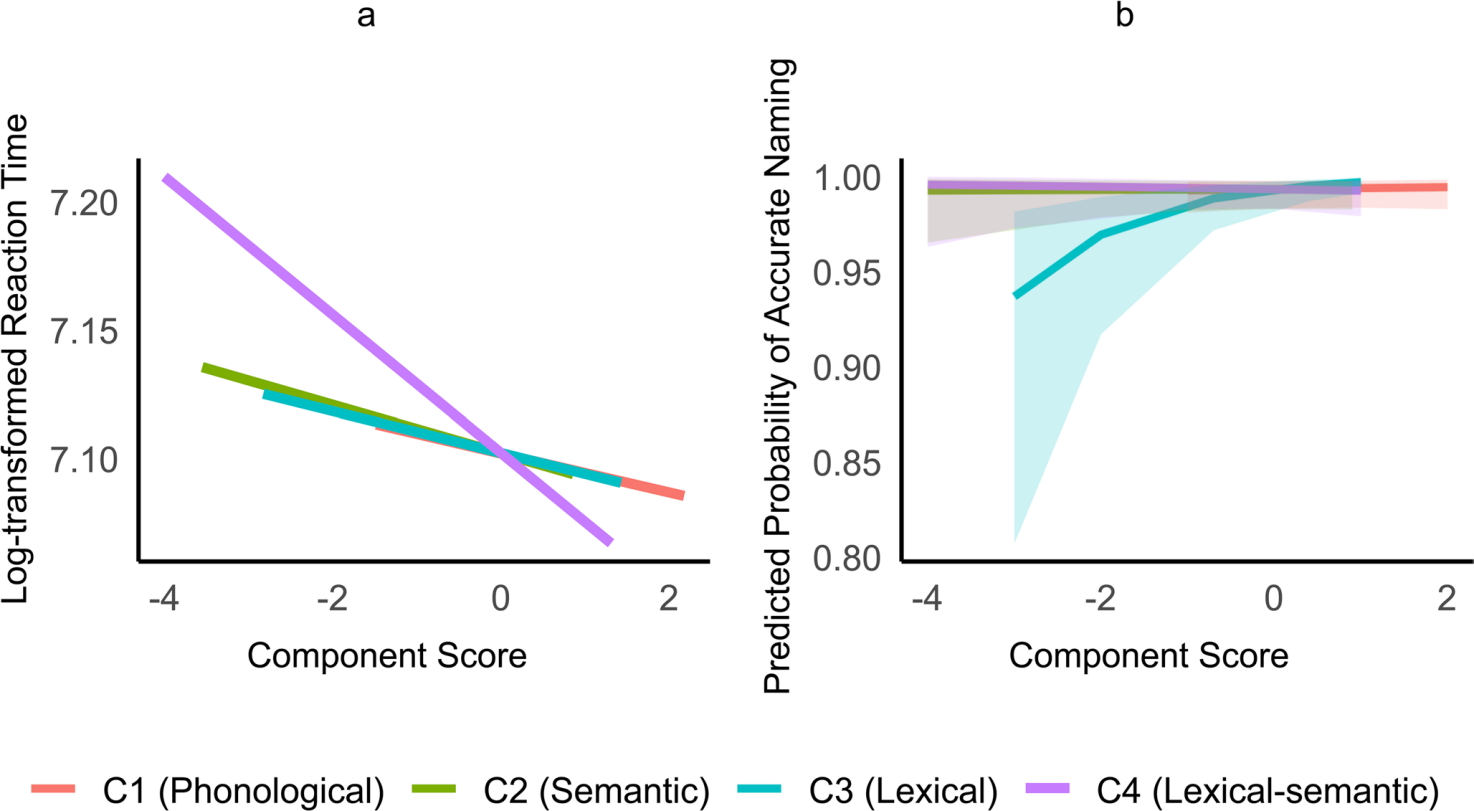
Regression of psycholinguistic components across naming speed (**a**) and accuracy (**b**)

The regression analyses for principal components and the outcome variables (i.e., RTs and accuracy) yielded the results shown in Table 7. C4 (a lexical-semantic component) significantly predicted naming reaction times, suggesting that the lexical-semantic properties of words drive naming speed in PFT patients (*p* = 0.025, see Fig. 6a). As for naming accuracy, the lexical component (C3) predicted the performance significantly (*p* < 0.001, see Fig. 6b). Reaction times were slightly shorter for the assessments done on the screen, which was expected because it took less time to swipe on the screen than to flip a page (*p* = 0.093). The interclass coefficient (ICC) of both models (i.e., 0.42 and 0.37) show that a substantial proportion of the total variance is attributable to differences between individuals.

**Table 7:** Mixed-effects regression results with reaction times for correct responses and principal components

Outcome variable	Predictors	Coefficients	CI	*p*
Fixed effects				
Reaction times	(Intercept)	7.37	7.10–7.65	**< 0.001**
	C1 (phonological)	−0.02	−0.05–0.00	0.062
	C2 (semantic)	−0.02	−0.04–0.01	0.132
	C3 (lexical)	−0.00	−0.03–0.03	0.797
	C4 (lexical-semantic)	−0.04	−0.08 – −0.01	**0.025**
	mode [screen]	−0.12	−0.26–0.02	0.093
	age	−0.03	−0.06 – −0.00	**0.037**
	Sex [female]	0.06	−0.16–0.28	0.606
Random effects				
	σ²	0.16		
	τ₀₀	0.11		
	ICC	0.42		
Fixed effects				
Accuracy	(Intercept)	1.71	0.39–9.86	0.503
	C1 (phonological)	1.09	0.81–1.46	0.577
	C2 (semantic)	1.02	0.75–1.40	0.877
	C3 (lexical)	2.12	1.50–3.00	**< 0.001**
	C4(lexical-semantic)	0.90	0.56–1.45	0.665
	age	1.60	1.29–1.99	**< 0.001**
	Sex [female]	1.94	0.53–7.11	0.318
Random effects				
	σ²	3.29		
	τ₀₀	1.90		
	ICC	0.37		

σ² = residual variance; τ₀₀ = random intercept variance; *ICC,* interclass correlation coefficient

### Change in Language Processing from Pre- to Post-surgery

For question 3, the study examined the change in how various linguistic levels predicted naming speed and accuracy across assessment points 1, preoperative, and 2, 1–4 weeks postoperative assessment. As shown in Table 8, none of the interactions between principal components representing the linguistic levels and the assessment moment were significant.

**Table 8:** Influence of principal components on naming reaction times and accuracy across assessment points

Outcome variable	Interactions	Estimates	CI	*p*
Reaction times	C1 × assessment point [2]	0.03	−0.02–0.08	0.196
	C2 × assessment point [2]	−0.01	−0.06–0.04	0.722
	C3 × assessment point [2]	−0.02	−0.08–0.03	0.426
	C4 × assessment point [2]	0.05	−0.02–0.12	0.192
		Odds ratios		
Accuracy	C1 × assessment point [2]	1.62	0.89–2.95	0.111
	C2 × assessment point [2]	0.76	0.39–1.48	0.421
	C3 × assessment point [2]	1.70	0.85–3.39	0.134
	C4 × assessment point [2]	0.92	0.35–2.41	0.866

C1 = Phonological component; C2 = Semantic component; C3 = Lexical component; C4 = Lexical-semantic component

## Discussion

We report that word-finding speed is slower after surgery, while accuracy did not change. Furthermore, word-finding speed is predicted by the lexical-semantic level (i.e., number of semantic features and word frequency), while the lexical-only component (age of acquisition, familiarity, and frequency) predicts word-finding accuracy. Lastly, there is no difference in how the above-mentioned variables predicted word-finding ability across assessment points.

### Developmental Performance Pattern on English Wordrace Data

Wordrace has not been standardized for English, so it is important to check whether the performance on Wordrace is consistent with the expected developmental trajectory of picture-naming ability. Previous literature on healthy populations has shown that word retrieval gets faster with increasing age as the lexical representations get stronger [48], and the current analysis found the same, i.e., a significant positive correlation between patients’ age and word-finding speed. Secondly, the analysis of naming accuracy revealed that age positively and significantly predicted the probability of producing a correct response. As evident from the plot in Fig. 4, this correlation linearly increased until around ten years of age and then remained constant afterward. This aligns with the literature confirming that naming accuracy on confrontational naming tests increases linearly in the initial years (from one to five years) and then remains constant in unimpaired populations [109].

### Word-Finding Ability Pre- and Post-surgery

The increase in naming reaction times post-surgery indicates a worsening of word-finding speed and aligns with previous studies that reported a decline in language function following surgical removal in supratentorial language-eloquent areas in adults [110, 111]. Particularly in the naming latency literature, decline in adult patients’ performance has been observed on the Boston Naming Test (BNT) after surgery compared to baseline performance before surgery [50]. Literature on cerebellar degeneration also shows that these adult patients present with significantly slowed word finding. The local involvement of the cerebellum in word-finding ability as evidenced by studies on lexical decision [11], phonemic fluency [13], and semantic tasks [14, 15], may be the primary reason for slow word-finding after surgery. While slow word finding does correlate with motor impairments, motor impairments are not the sole underlying cause of this speed reduction, as the severed connections of the cerebellum with the cerebrum may also explain word-finding difficulties [112]. Therefore, surgery might increase the lesion size compared to the lesion due to the tumour only, thus resulting in slowed word finding after surgery. It is also important to note that since we used the same test at both assessment points, practice effects might have attenuated the observed decline in word-finding speed and possibly reduced sensitivity to detect a decline in accuracy.

### Language Processing Underlying Word-Finding Ability

Effects of word properties on language performance were investigated as these may be used to characterise the nature of language impairments in clinical populations [56, 62], but these inferences are based on the observation that the same variables affect performance in healthy individuals [60, 62, 65]. The significant effect of the lexical-semantic linguistic level on word-finding speed could thus signal the effective use of lexical and semantic resources to achieve accurate naming in patients. Future studies including controls can test if this effect is atypical in any way (larger or smaller than in controls). The primary implications of these findings are, therefore, hypothesis generation: in future studies, atypical effects compared to controls could signal impairment, in line with the studies reviewed by [4], where joint impairments of lexical and semantic processing have been reported in PFT patients in 26% of the studies that tested for it through expressive vocabulary or naming tasks. Regarding the psycholinguistic nature of impairment in patients undergoing cerebellar tumour surgery, the lexical level was found to be impaired in three out of twelve patients [92].

The lexical level (C3) also significantly predicted response accuracy. This level consisted of frequency, age of acquisition, and familiarity. Words frequently occurring in the corpus, more familiar to people due to exposure, and learned early on in the acquisition process are more likely to be retrieved correctly in healthy individuals and as such their effect could signal effective lexical processing supporting fast naming, while atypical effects of these variables in patients may signal lexical impairment [78, 80, 113].

In the current study, other linguistic levels did not significantly predict word retrieval speed, which can be attributed to the task’s easy nature when considering the participants’ age. The pictures included in the task do not vary much in complexity and are of highly concrete, imageable, and frequent nouns, which may result in relatively easier access to the representations at levels such as semantics and phonology. As noted in a previous study, picture-based tasks constrain the range of possible verbal responses and psycholinguistic variability, especially when stimuli are designed for use with children [92]. Moreover, we had expected a significant effect of the mainly semantic level (C2) in driving word-finding ability, but did not find such an effect. It could be that in order to find such an effect, the items of the test need to be carefully controlled for semantic similarity and complexity, which could be a direction for future studies. Moreover, the effect seen at the lexical-semantic level may also have trickled down from the semantic level. For instance, competition between semantic neighbours may result in difficulty in selecting the target concept at the semantic level, leading to the same sort of difficulty in lexical selection [56].

Whether patients show an atypical effect of these variables (which is a clear indication of impairment) can only be determined by future studies including healthy controls. However, if the influence of variables changes in patients as a consequence of surgery, along with a change in performance, then this could signal a change into impaired processing at a certain level of processing, as evaluated in the next section.

### Language Processing Pre- and Post-surgery

The way psycholinguistic levels affect word-finding ability did not change significantly in our PFT sample across the two assessment points. The lack of evidence for additional types of impairment may reflect that the anatomical substrates affected by surgery are mainly determined by the location of the tumour [114], diaschisis via tumour compression [52, 53], and/or supratentorial disruption due to hydrocephalus [54], and thus relevant impairments are already present before surgery, albeit in a less severe form [95]. This would imply that different kinds of tumour could lead to a distinct pattern: for example, in adult patients with supratentorial tumours, tumour grade is an important factor to consider when investigating the change in performance pre- and post-surgery since patients with low-grade glioma tend to have already gone through neuroplastic changes due to the slow progression of the tumour and would be expected not to show as much decline after surgery [50]. On the other hand, high-grade glioma patients show more drastic changes right after surgery because the lesion affects parts of their eloquent brain networks adversely due to the functions not having enough time to relocate to a different brain region.

Another hypothesis for a decay in naming speed in the absence of evidence for changes within the language processing system, is that naming speed may be reduced in the context of a more general cognitive mechanism, such as processing speed, which critically interacts with other cognitive functions (e.g., language), and is shown to be frequently impaired in children with posterior fossa tumours [115]. Although most studies assessing processing speed have compared patients with and without administration of radiotherapy, one study showed that PFT patients who did not undergo adjuvant radiotherapy also presented with slower information processing speed compared to a non-CNS tumour control group [33]. This could explain a general decay in language performance (i.e., specifically word-finding speed in the current study), which is nonspecific to any particular linguistic level.

Future studies may also focus on studying language ability at later time points and on the effects that adjuvant radiotherapy may have in leading to additional linguistic impairments or changing the psycholinguistic nature of word-finding ability. Such a pattern has been shown by in an investigation of rapid picture naming as part of information processing speed which revealed that the difference in processing speed was more pronounced after the irradiation therapy [116].

### Limitations and Future Directions

The current study did not have control data to compare the word-finding ability of PFT patients. This limited the interpretation of the linguistic levels that significantly impacted accuracy and reaction times in terms of their contribution to the impairment status. Future studies should recruit age-matched controls and replicate the current study to find if their word-finding ability is influenced by the same linguistic levels as the PFT sample. The approach used in previous studies for narrative data can also be employed to check whether principal component analysis with the psycholinguistic variables and word-finding ability helps differentiate between controls and patients [92]. Moreover, a larger sample size is required to establish whether the effect of psycholinguistic properties on word-finding performance changes due to surgery. With the limited sample size of the current study, we can merely read the null interactions as absence of evidence for such a change.

Wordrace needs to be standardized in English and other languages, adding to the construct validity of the test. An even better approach would be to rethink the test design, balancing items on visual complexity and psycholinguistic properties of the target words. Potentially significant factors, such as the selection of stimuli and their order, are usually overlooked in picture naming tests [117]. For instance, the effect of semantic categories can obscure performance [118].

Lastly, more assessment points should be investigated to examine the trajectory of word-finding ability over time after surgery in terms of improvement or worsening. As reported in a recent systematic review [4], some studies that tested language ability longitudinally in the PFT sample, revealed inconsistent results, with some finding no impairment shortly after surgery but finding phonological and pragmatic deficits at one-year follow-up [119], while others reported persistent lexical-semantic difficulties at all assessment points after surgery [39]. A longitudinal study would also help pinpoint how radiotherapy affects word retrieval abilities and how this evolution might differ depending on specific patient characteristics such as age, sex, language background (i.e., monolingual/bilingual), and/or socioeconomic status.

## Conclusion

In conclusion, this study provides important insights into the relationship between linguistic processing levels and word-finding ability in patients with posterior fossa tumours (PFT), both before and after surgical intervention. By examining the influence of psycholinguistic levels on word-finding accuracy and speed, we found that lexical-semantic processing significantly predicted word-finding speed, while only lexical processing predicted accuracy. Moreover, the observed decline in word-finding speed post-surgery suggests a deterioration in lexical retrieval, consistent with previous studies on language function post-surgery in language-eloquent areas. However, the stability of the predictive influence of psycholinguistic levels across assessment points indicates that while surgery may exacerbate existing impairments, it does not necessarily alter the underlying linguistic processing mechanisms, and we cannot ascribe the decay in language ability specifically to lexical processing. Rather, this may be explained by more general cognitive processing limitations (e.g., in processing speed). Importantly, method validation confirmed the Wordrace test’s construct validity, aligning with known developmental patterns in naming abilities, thus establishing its utility for assessing word-finding performance.

Future research should address the limitations identified, such as the lack of control data and the need for a standardized word-finding test. Additionally, longitudinal studies with more assessment points would provide a clearer trajectory of word-finding ability over time, considering factors like radiotherapy and patient characteristics. Given that previous studies show that word finding may be impaired long after surgery, future studies may focus on how psycholinguistic processing skills underlying word finding may change in response to radio- and/or chemotherapy as shown in other cognitive skills [120] or how intervention in speech-language therapy may shape psycholinguistic processing in this population, as shown in other clinical groups [121]. Moreover, language performance should be analysed in relation to important clinical factors such as the type and site of the tumour, using techniques such as voxel-based lesion symptom mapping, which can potentially provide a clearer understanding of the neural correlates of language impairments and also identify the posterior fossa structures that are important for language functions such as word finding. These steps will further help us understand the complex dynamics of language processing in PFT patients, ultimately guiding better clinical practices and rehabilitation strategies.

## Data Availability

The data used in this research are part of the European Cerebellar Mutism Study and were shared with researchers from the University of Groningen for the purposes of this study. Data requests should be sent to the PI of the European Cerebellar Mutism Study (Rene.Mathiasen@regionh.dk).

## Appendix

**Table 9:** Previous studies testing naming abilities in the PFT population

No.	Study	No. of patients	Naming test	Outcome	Cross-sectional/longitudinal	Pre-operative testing
1	Cámara et al., 2020 [26]	36(+34 controls)	KABC-II Expressive Vocabulary	Naming was significantly impaired in patients vs controls (p=0.038) and in the pCMS group vs non-pCMS (p=0.049)	Cross-sectional	No
2	Docking et al., 2016 [31]	10+(10 controls)	HPNT	Scores not compared to controls (only used to examine the relationship between narrative performance and structural language)	Cross-sectional	No
3	Grieco et al., 2020 [32]	36	EOWPVT-4	Performance of PFT patients with CMS was significantly worse than ones without CMS but not below the normal range at first assessment point.	Longitudinal (baseline assessment at the initiation of proton radiotherapy and follow-up after one year)	No
4	Mabbott et al., 2008 [33]	64	Rapid Picture Naming Task	Performance on rapid naming was significantly (p<.05) worse in patients with PFT surgery who also underwent radiotherapy than controls but not for patients with surgery alone.	Cross-sectional	No
5	O’Neil et al., 2020 [34]	24	Vocabulary subtest of WISC-IV	Scores from vocabulary tests were combined with other measures of intelligence for analysis so no definite outcome regarding expressive vocabulary	Cross-sectional	No
6	Pletschko et al., 2018 [35]	14(+14 controls)	Vocabulary subtest of Wechsler Intelligence Scale for adults/children depending on participants’ ages	Vocabulary scores of patients were significantly (p<0.01) lower than high-achieving controls but not average-performing controls	Cross-sectional	No
7	Steinlin et al., 2003 [36]	24	Token test (although this is a comprehension test, this study used it for naming - they don’t give additional information about the way this was administered)	No impairment in naming compared to norms	Cross-sectional	No
8	Di Rocco et al., 2011 [37]	23	Naming section of FLT and BNT (for children over 5 years)	Individual analysis = 10 out of 23 children presented with pathological naming	Cross-sectional	Yes
9	Richter et al., 2005 [28]	11(+27 controls)	Naming condition (block) of the verb generation task	No significant difference between reaction times of controls and patients for naming	Cross-sectional	No
10	Grill et al., 1999 [38]	19	Vocabulary subtest of KABC	No control group for comparison but found significantly poor vocabulary (p=0.03) for group with higher irradiation dose (35 Gy) than the one with lower dose (25 Gy)	Cross-sectional	No
11	De Smet et al., 2009 [39]	5	Dutch language battery for children (Taaltest voor Kinderen)	Reports of individual patients with problems reported in expressive language abilities and one patient specifically presented severe word-finding deficit	Longitudinal (2 months after surgery and 2 years after surgery on average)	No
12	De Witte et al., 2017 [40]	1	BNT	Impaired naming; BNT score = 38/60 (z= -2.22)	Cross-sectional	No
13	Levisohn et al., 2000 [41]	19	depending on the age of patients: Naming vocabulary subtest of the DAS, EOWPVT, or BNT	37% of the patients sample presented with expressive language impairment with an additional four patients with exclusive word-finding difficulties	Cross-sectional	No
14	Murdoch et al., 2004 [42]	12 (+12 controls)	HPNT	Not impaired; no patient scored below 93/100 except one who scored 55/100 (below the normal range)	Cross-sectional	No
15	Frank et al., 2007 [43]	9 (+11 controls)	Naming block within the verb generation task	Longer reaction times for patients with cerebellar lesions but response accuracy was not affected	Longitudinal (before and after surgery)	Yes
16	Kristiansen et al., 2021 [44]	20	BNT	Patients performed significantly below the norm (mean score = 49.8 and p =.049)	Cross-sectional	No
17	Aarsen et al., 2009 [45]	32	BNT	Patients had significantly low score compared to normative data (p <.01)	Cross-sectional	No
18	Riva & Giorgi, 2000 [46]	26	BNT	Less pronounced impairment (SD = -1.5)	Cross-sectional	No
19	Zilli et al., 2021 [47]	7	BVL 4-12	Naming not impaired	Cross-sectional	No
20	Persson et al., 2024 [48]	148	Wordrace	Slow word-finding = 37%Inaccurate word-finding = 24%Slow and inaccurate word-finding = 16%	Cross-sectional	Yes
21	Persson et al., 2025 [49]	184 (total)Swedish subset for control comparison (n=49)	Wordrace	Impaired word-finding speed, with 95% performing ≥2 SDs slower than norms postoperatively.accuracy was relatively preserved, with only 16% scoring ≥1 SD below norms and 5% showing serious impairmentPreoperatively, total test times approximately twice as long as norms, while accuracy was mainly intact.	Longitudinal (before and after surgery)	yes

*KABC,* Kaufman Assessment Battery for Children; *pCMS,* Postoperative Cerebellar Mutism Syndrome; *HPNT,* Hundred Picture Naming Test; *EOWPVT,* Expressive One-Word Picture Vocabulary Test; *FLT,* First Language Test; *BNT,* Boston Naming Test; *WASI,* Wechsler Abbreviated Scale of Intelligence; *DAS,* Differential Abilities Scale; *SD,* Standard Dveiation; *BVL,* Battery for the assessment of language in children for 4-12 years
